# 17β‐estradiol promotes the progression of temporomandibular joint osteoarthritis by regulating the FTO/IGF2BP1/m6A‐NLRC5 axis

**DOI:** 10.1002/iid3.1361

**Published:** 2024-08-02

**Authors:** Xintong Xue, Changyi Li, Shuang Chen, Yan Zheng, Fan Zhang, Yan Xu

**Affiliations:** ^1^ Department of Orthodontics, Shanghai Stomatological Hospital & School of Stomatology Fudan University Shanghai China; ^2^ Shanghai Key Laboratory of Craniomaxillofacial Development and Diseases Fudan University Shanghai China; ^3^ Department of Endodontics, Shanghai Stomatological Hospital & School of Stomatology Fudan University Shanghai China; ^4^ Department of Prosthodontics, Shanghai Stomatological Hospital & School of Stomatology Fudan University Shanghai China; ^5^ Department of Implantology, Shanghai Stomatological Hospital & School of Stomatology Fudan University Shanghai China

**Keywords:** 17β‐estradiol, fibroblast‐like synoviocytes, FTO, m6A methylation, NLRC5, temporomandibular joint osteoarthritis

## Abstract

**Background:**

Temporomandibular joint osteoarthritis (TMJOA) is a degenerative cartilage disease. 17β‐estradiol (E2) aggravates the pathological process of TMJOA; however, the mechanisms of its action have not been elucidated. Thus, we investigate the influence of E2 on the cellular biological behaviors of synoviocytes and the molecular mechanisms.

**Methods:**

Primary fibroblast‐like synoviocytes (FLSs) isolated from rats were treated with TNF‐α to establish cell model, and phenotypes were evaluated using cell counting kit‐8, EdU, Tanswell, enzyme‐linked immunosorbent assay, and quantitative real‐time PCR (qPCR). The underlying mechanism of E2, FTO‐mediated NLRC5 m6A methylation, was assessed using microarray, methylated RNA immunoprecipitation, qPCR, and western blot. Moreover, TMJOA‐like rat model was established by intra‐articular injection of monosodium iodoacetate (MIA), and bone morphology and pathology were assessed using micro‐CT and H&E staining.

**Results:**

The results illustrated that E2 facilitated the proliferation, migration, invasion, and inflammation of TNF‐α‐treated FLSs. FTO expression was downregulated in TMJOA and was reduced by E2 in FLSs. Knockdown of FTO promoted m6A methylation of NLRC5 and enhanced NLRC5 stability by IGF2BP1 recognition. Moreover, E2 promoted TMJ pathology and condyle remodeling, and increased bone mineral density and trabecular bone volume fraction, which was rescued by NLRC5 knockdown.

**Conclusion:**

E2 promoted the progression of TMJOA.

## Graphical abstract

E2 aggravates MIA‐induced temporomandibular joint osteoarthritis‐like changes by regulating NLRC5.

## 1 INTRODUCTION

Temporomandibular joint osteoarthritis (TMJOA) is a degenerative cartilage disease that will induce pain in the TMJ.^1^, ^2^ TMJOA presents with limited mouth opening, abnormal jaw movement, tinnitus, and pain,^3^ affecting approximately 18%–85% of patients with temporomandibular disorders.^4^ It is characterized by cartilage degeneration, TMJ remodeling, and synovitis.^5^ TMJ is a synovial joint that contains a mandibular condyle, glenoid fossa, and articular disc.^6^ The TMJ ensures the balance of chewing, swallowing, and other functions, and its long‐term or excessive load causes the dysfunction of TMJ.^7^ Although the treatment technology of TMJOA is developing, and the existing therapy can effectively relieve the symptoms; however, the long‐term treatment effect is not satisfactory.^5^ Thus, it is urgent to clarify the pathogenesis of TMJOA and explore a more effective strategy.

Synoviocytes are located in the lining layer of the synovium and are composed of macrophage‐like synoviocytes (MLSs) and fibroblast‐like synoviocytes (FLSs).^8^ Previous studies have reported that FLSs are involved in the pathogenesis of rheumatoid arthritis, osteoarthritis, and TMJOA.^9^, ^10^, ^11^ It has been reported that TMJ synoviocytes stimulated by TNF‐α and IL‐1β can produce multiple biologic factors and promote proliferation and invasion properties, thereby facilitating cartilage damage.^12^, ^13^ Therefore, studying the function of FLSs is helpful to elucidate the pathogenesis of TMJOA, and TNF‐α‐induced FLSs is a suitable cell model to simulate FLSs in TMJOA state.

Since the incidence of TMJOA is higher in women than in men,^14^ it is speculated that estrogen may play a key role in TMJOA. 17β‐estradiol (E2) is a natural estrogen, widely present in the environment.^15^ It has been revealed that E2 is an endocrine‐disrupting chemical that interferes with normal hormonal action in humans and animals and is associated with abnormal growth, metabolism, and reproduction system.^16^ Estrogen has both pro‐inflammatory and anti‐inflammatory effects in immune regulation, and these contradictory effects depend on different immune stimuli, cell types, pathological processes, administration time, the concentration of use, and so forth.^17^ Recent studies have shown that E2 induces the pathological progression of TMJOA.^18^, ^19^ However, the effects of E2 on cell behaviors in TMJOA and its potential regulatory mechanism are not discussed.

For this purpose, we evaluated whether E2 regulates the growth, metastasis, and inflammatory phenotype of synoviocytes in this study. Moreover, we explored the underlying mechanism of E2 functions in TMJOA. The findings provide a deeper theoretical basis for E2 to promote the development of TMJOA.

## 2 MATERIALS AND METHODS

### 2.1 Materials

Trypsin (T4799), paraformaldehyde (95%, 158127), crystal violet (C0775), hematoxylin (H3136), eosin (E4009), 4′,6‐Diamidine‐2′‐phenylindole dihydrochloride (DAPI; 10236276001), E2 (3301), actinomycin D (ActD; SBR00013), and monosodium iodoacetate (MIA; ≥98%, I2512) were purchased from Sigma‐Aldrich. Dulbecco's modified eagle's medium (DMEM; A1443001), fetal bovine serum (FBS; 10100147C), glutamine (25030149), and penicillin/streptomycin (15140122) were purchased from Gibco. Recombinant rat TNF‐α protein (HY‐P7108) was acquired from MedChemExpress. Trizol (15596026) and Lipofectamine 2000 (11668500) were obtained from Invitrogen. The cell counting kit‐8 (CCK‐8; A311‐01), HiScript Q RT SuperMix (R123‐01), SYBR Color qPCR Master Mix (Q411‐02), BCA protein quantification kit (E112‐01), and ECL chemiluminescence kit (E411‐04) were purchased from Vazyme. Antibody diluents (A1800 and A1820) were purchased from Solarbio. The EdU proliferation kit (ab222421) was purchased from Abcam. Rat IL‐1β ELISA kit (J22380) and IL‐6 ELISA kit (J22434) were obtained from GILED Biotechnology. The RNA binding protein immunoprecipitation (RIP) kit (KT102‐01) was obtained from Gzscbio. The EpiQuik m6A RNA methylation quantification kit (P‐9005) and EpiQuik MeRIP kit (P‐9018‐24) were acquired from Epigentek. Matrigel (354234) was purchased from Corning.

### 2.2 Animals

Sprague‐Dawley (SD) rats (female, 280–300 g; Vital River) were kept in the specific pathogen‐free conditions of 22 ± 1°C, 12 h light/dark cycle, 50%–60% humidity, and free assessment of food and water. The animal study was approved by the ethics committee of Shanghai Stomatological Hospital & School of Stomatology, Fudan University and was performed according to the ARRIVE guidelines.

### 2.3 FLSs isolation

FLSs were isolated from the synovial tissues of TMJ of rats as previously described.^20^ The tissues were cut into small pieces and digested to cell suspension using 0.25% trypsin. Then, the suspension was incubated in DMEM supplemented with 10% FBS, 2 mM glutamine, and 1% penicillin/streptomycin in T25 culture flasks at 37°C with 5% CO_2_. FLSs were isolated using differential adhesion, cultured to 100% confluence, and passaged. FLSs were used for subsequent experiments in passages 3–6.

### 2.4 Cell treatment

To generate the cell model, FLSs were exposed to 10 ng/mL of recombinant TNF‐α protein for 24 h. To explore the effect of E2 on cellular behaviors, E2 (100 nM) was used to treat FLSs for 24 h.^21^

### 2.5 CCK‐8

FLSs (100 μL, 2000 cells) were seeded in 96‐well plates and cultured for 24 h. CCK‐8 solution (10 μL) was added to plates and incubated for 1 h. The absorption value at 450 nm was detected by a microplate reader.

### 2.6 EdU assay

An EdU proliferation kit was used to analyze cell proliferation. FLSs were incubated with the culture medium until 60%–70% confluence and labeled with 10 μM EdU solution for 4 h. Then, the cells were fixed with 200 μL 1 × fixative solution for 20 min and permeabilized with 200 μL 1 × permeabilization buffer for 30 min. FLSs were incubated with 100 μL EdU reaction solution for 30 min in the dark. DNA was stained using 100 μL 5 μg/mL DAPI for 30 min. The results were visualized using a fluorescence microscope equipped with filter for excitation/emission wavelengths of 649/664 nm (magnification: ×20).

### 2.7 Transwell assay

Cell migration was assessed using 24‐well Transwell chambers (8 μm pore size). Cell invasion was analyzed using the Transwell chambers precoated with Matrigel. Then, Transwell assay was performed as previously described with minor changes.^22^ Briefly, serum‐free medium containing FLSs (200 μL, 5 × 10^4^ cells) was added to the upper chambers. The complete medium (500 μL) was added to the bottom chambers. After 24 h of incubation at 37°C with 5% CO_2_, the cells on the lower surface of the membranes were immobilized with 4% paraformaldehyde for 20 min and stained with 0.1% crystal violet for 15 min. The stained cells were visualized under an inverted light microscope at the magnification of ×20, and the number of migrated or invaded cells was counted using five randomly fields.

### 2.8 Enzyme‐linked immunosorbent assay (ELISA)

The levels of IL‐1β and IL‐6 were detected using the rat IL‐1β and IL‐6 ELISA kits respectively according to the manufacturer's instructions. Briefly, the supernatant from cells was collected and centrifuged at 300*g* at 4°C for 10 min to remove cell debris. The sample (10 μL) was diluted by 40 μL diluent and incubated with 100 μL enzyme‐labeled reagent at 37°C for 60 min. After washing, the sample was incubated with color developing reagent at 37°C for 15 min. Following adding 50 μL stop buffer, the absorbance was measured at 450 nm using a microplate reader.

### 2.9 Quantitative real‐time PCR (qPCR)

Total RNA in FLSs was isolated using the Trizol reagent. cDNA reverse‐transcribed from RNA was synthesized using the HiScript Q RT SuperMix. Quantitative PCR was performed using the SYBR Color qPCR Master Mix on an Applied Biosystems 7500 system. Results were calculated by the $2^{-\Delta \Delta C_{t}}$ method based on the internal control GAPDH. The primer sequences are listed in Table 1.

**Table 1:** Primer sequences used in qPCR.

Name	Forward (5′‐3′)	Reverse (5′‐3′)
METTL3	CCTAAGCCCAGCACAACG	GAGATGGCAAGACGGATC
METTL14	CGCAGCACCTCGGTCATT	TCGCTTCACGGTTCCTTT
WTAP	ATGGCACGGGATGAGTTA	CCTGCTGTTGCTGCTTTA
FTO	CCGTGGAACAAAGGAGTG	CAAAGGGCAGAGGCATAG
ALKBH5	CTTTAGCGACTCGGCACTT	CTCATCAGCAGCATACCCAC
NLRC5	ACCTGGGAGTCTTTCATCTA	CTGCTTCCTGCTGTGCTT
MMP3	CTGAAGATGACAGGGAAGC	CTGGAGAATGTGAGTGGG
MMP9	ACCGCTATGGTTACACTCG	TCTCGCTGTCCAGCTCAC
GAPDH	GCAAGTTCAACGGCACAG	CGCCAGTAGACTCCACGAC

### 2.10 Microarray

The original microarray data set GSE205389 was downloaded from the GEO database. The differentially expressed genes were predicted using the R language and were defined as |log(FC)|>1 and *p* < .05. Differential expression of m6A methylation‐related enzymes was shown.

### 2.11 Detection of m6A level

An EpiQuik m6A RNA methylation quantification kit was used to detect m6A methylation levels in FLSs according to the manufacturer's protocol. In brief, total RNA was isolated from FLSs using Trizol. The RNA was incubated with negative and positive controls at 37°C for 90 min. The samples were incubated with capture antibody (60 min), detection antibody (30 min), and enhancer solution (30 min) for specified times successively, and washed with washing solution three times after each incubation step. Developer solution was added for incubation at room temperature away from light. After stopping the reaction, the absorbance was read on a microplate reader at 450 nm.

### 2.12 Western blot

Total protein was extracted from FLSs using RIPA buffer. Protein concentration was measured by a BCA kit. Protein (30 μg) was resolved using 10% SDS‐PAGE, followed by transfer to PVDF membranes. The samples were blocked using 5% skim milk for 10 min and incubated with the primary antibodies against NLRC5 (ab105411; Abcam), FTO (ab126605; Abcam) and GAPDH (ab9485; Abcam) at 4°C overnight. The secondary antibody (ab97051; Abcam) was incubated with the membranes at room temperature for 1 h. All antibodies were diluted using corresponding antibody diluents. The bands were developed using the ECL kit. Gray analysis was performed using the ImageJ 1.8.0 software.

### 2.13 Cell transfection

FTO overexpression vector, IGF2BP1 overexpression vector, control vector, shRNA (sh)‐FTO, sh‐NLRC5, sh‐IGF2BP1, and sh‐negative control (sh‐NC) were synthesized by GenePharma. FLSs were seeded into six‐well plates at the concentration of 2 × 10^5^ cells/well and grew over 70% cell confluence. The vectors/opti‐MEM dilution (4/250 μL) was mixed with lipofectamine 2000/opti‐MEM dilution (10/250 μL) for 20 min. Then, FLSs were incubated with the mixture at 37°C for 6 h, and the serum‐free medium was replaced by complete medium. Transfection was performed for 48 h.

### 2.14 Methylated RNA immunoprecipitation (MeRIP)

The NLRC5 m6A levels were detected by an EpiQuik MeRIP kit following the manufacturer's instructions. Total RNA was incubated with immune capture buffer, affinity beads, and m6A antibody or nonimmune IgG at room temperature for 90 min. After removing the supernatant, the beads were washed with wash buffer and protein digestion buffer. RNA binding beads were collected to elute RNA. The expression of IGF2BP1 was measured using qPCR.

### 2.15 ActD treatment

Transfected FLSs were treated with 5 μg/mL AtcD for 0, 3, 6, and 9 h. At the pointed times, the mRNA expression of NLRC5 was detected using qPCR.

### 2.16 RIP

The binding relationship between m6A readers and NLRC5 was analyzed using the RIP kit based on the manufacturer's protocol. FLSs were lysed using 1 mL cell lysis buffer for 2 h and centrifuged at 4000*g* for 15 min. Protein G/A magnetic beads were resuspended and precoated with IgG or antibodies against m6A “readers” for 8 h. Cell lysate was incubated with magnetic bead‐antibody mixture at 4°C overnight. The magnetic beads were washed and RNA was purified. Total RNA was isolated and NLRC5 expression was measured using qPCR.

### 2.17 Animal model establishment

Female SD rats were randomly divided into three groups: sham, MIA+E2, MIA+E2+sh‐NLRC5 (*n* = 6/group). Intra‐articular injection of MIA was used to generate the TMJOA‐like rat model.^23^ MIA (0.5 mg) was dissolved in normal saline (50 μL) and injected into the left TMJ below the cheekbone. The rats in the sham group were injected with an equal amount of normal saline at the same position. One week before injection, E2 (20 μg) was intraperitoneally injected into the rats. One week after MIA injection, adenovirus targeting sh‐NLRC5 were injected into the left TMJ cavity at the same location as the MIA injection for 3 weeks, twice per week. Four weeks after MIA injection, all rats were killed by overdose injection of pentobarbital. The TMJ was acquired from each rat and fixed in 4% paraformaldehyde for bone morphological analysis using microcomputed tomography (micro‐CT). Additionally, the TMJ was demineralized with 15% EDTA after fixation and was used to prepare 5 μm sagittal paraffin sections.

### 2.18 H&E staining assay

Paraffin sections were dewaxed using xylene and rehydrated using ethanol. Then, the slides were incubated with hematoxylin for 5 min. The sections were incubated with 1% alcohol hydrochloric acid and 0.6% ammonia to remove excrescent hematoxylin and make the nuclei appear blue. The slides were incubated with eosin for 3 min. The results were visualized under a light microscope at the magnification of ×50.

### 2.19 Micro‐CT

Bone microarchitecture of condyles was visualized by x‐ray radiographs using the micro‐CT imaging system (Inveon; Siemens). Bone structure parameters including bone mineral density (BMD) and trabecular bone volume fraction (BV/TV) were acquired from micro‐CT scanning.

### 2.20 Safranin O staining

Paraffin sections were conventionally dewaxed and rehydrated, followed by stained with Weigert stain solution. After washing with water, Acidic ethanol differentiation solution was added to incubate with the sections for 15 s. Then, the samples were dip‐dyed in solid green solution for 5 min, washed with distilled water, and dip‐dyed in Safranin O solution for 2 min. Acetic acid solution was used to wash the sections to remove residual solid green. After dehydration, permeabilization, and sealing, the sections were observed under a light microscope.

### 2.21 Statistical analysis

Data from three independent replicates were analyzed using the GraphPad Prism 7 software and presented as the mean ± standard deviation. Comparisons between two groups or among multiple groups were analyzed by Student's *t* test or one‐way analysis of variance, respectively. *p* < .05 was statistically significant.

## 3 RESULTS

### 3.1 TNF‐α induces the proliferation, migration, invasion, inflammation, and extracellular matrix degradation of FLSs

**Figure 1:**
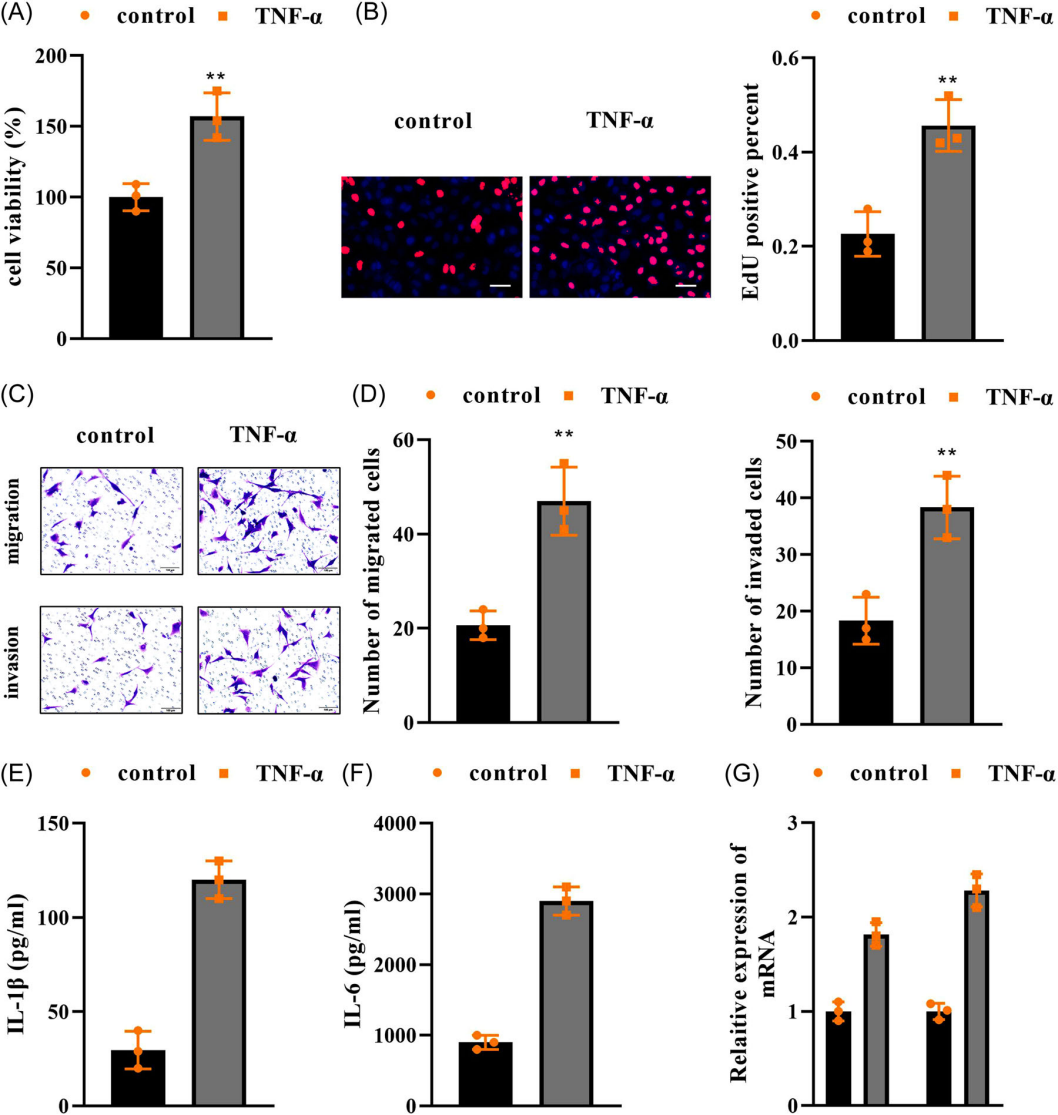
TNF‐α induces the proliferation, migration, invasion, inflammation, and extracellular matrix degradation of FLSs. FLSs were stimulated with TNF‐α, (A) CCK‐8 and (B) EdU assays determined cell proliferation capability, scale bar: 100 μm, magnification: ×20; (C and D) cell migration and invasion were assessed using Transwell assay, scale bar: 100 μm, magnification: ×20; (E) IL‐1β and (F) IL‐6 levels were detected by ELISA; (G) mRNA expression of MMP3 and MMP8 was measured by qPCR. *n* = 3. **p < .01. FLSs, fibroblast‐like synoviocytes.

To assess cellular biological behaviors, we used TNF‐α to treat FLSs to generate the cell model of TMJOA. The results showed that TNF‐α promoted cell viability and increased EdU‐positive cells, suggesting cell proliferation was promoted (Figure 1A,B). The migration and invasion, evaluated using Transwell assay, were facilitated by TNF‐α (Figure 1C,D). Besides, TNF‐α increased IL‐1β and IL‐6 levels, and mRNA expression of MMP3 and MMP9 (Figure 1E–G). The results indicated that the cell model was successfully established.

### 3.2 E2 promoted the biological functions of TNF‐α‐treated FLSs

**Figure 2:**
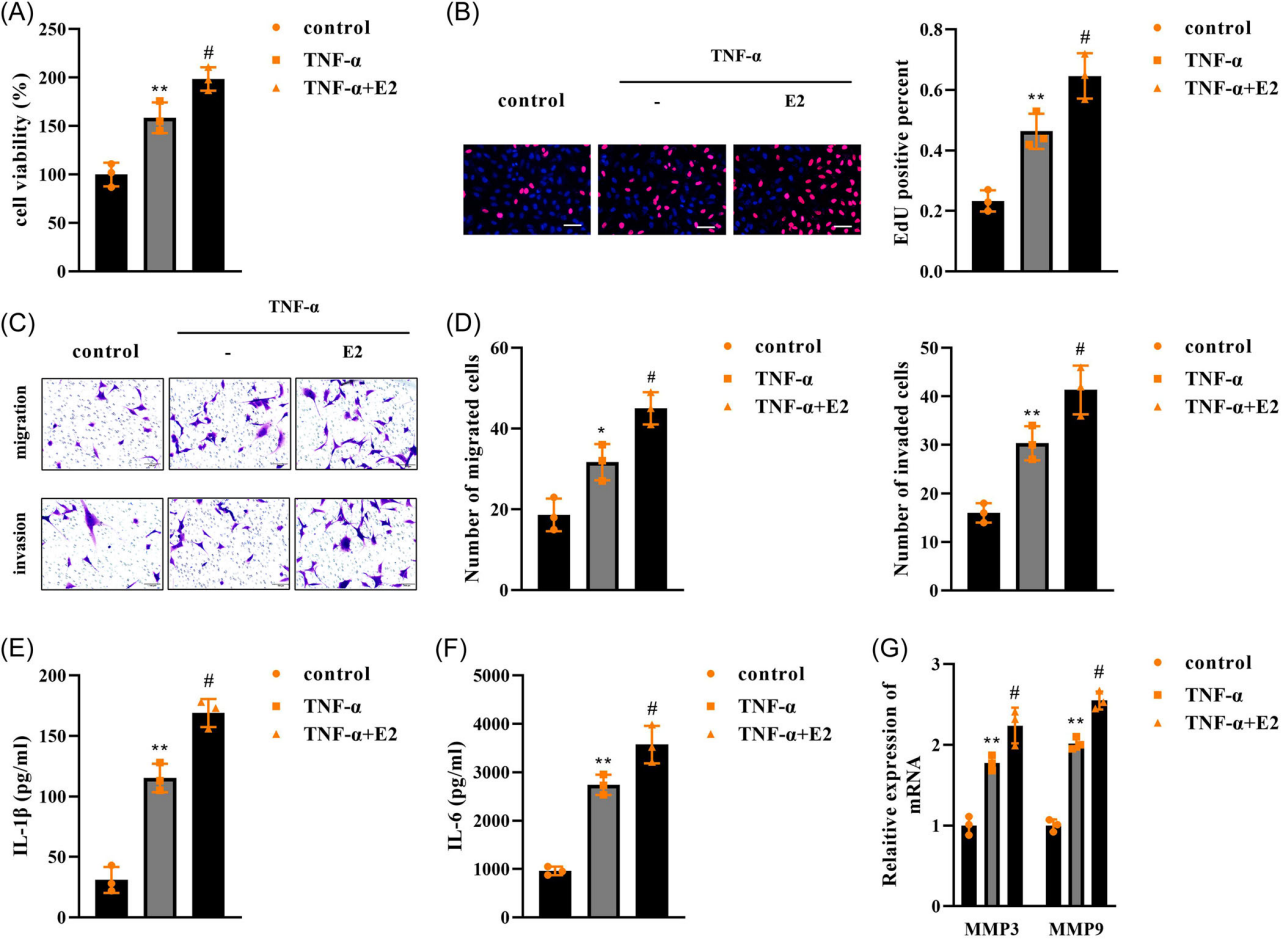
E2 promoted the biological functions of TNF‐α‐treated FLSs. FLSs were exposed to TNF‐α and E2, (A) cell viability was evaluated by CCK‐8; (B) cell proliferation was assessed using EdU assay, scale bar: 100 μm, magnification: ×20; (C and D) migration and invasion were measured using Transwell assay, scale bar: 100 μm, magnification: ×20; ELISA determined (E) IL‐1β and (F) IL‐6 levels; (G) MMP3 and MMP9 expression was detected by qPCR. *n* = 3. **p < .01. #*p* < .01. FLSs, fibroblast‐like synoviocytes.

The effects of E2 were further analyzed. We found that E2 further promoted the proliferation of FLSs stimulated with TNF‐α (Figure 2A,B). Additionally, migration and invasion of TNF‐α‐treated FLSs were facilitated by E2 (Figure 2C,D). Moreover, IL‐1β (Figure 2E), IL‐6 (Figure 2F), MMP3, and MMP9 (Figure 2G) levels were increased by TNF‐α, which were further increased by E2 treatment.

### 3.3 E2 downregulated FTO levels

**Figure 3:**
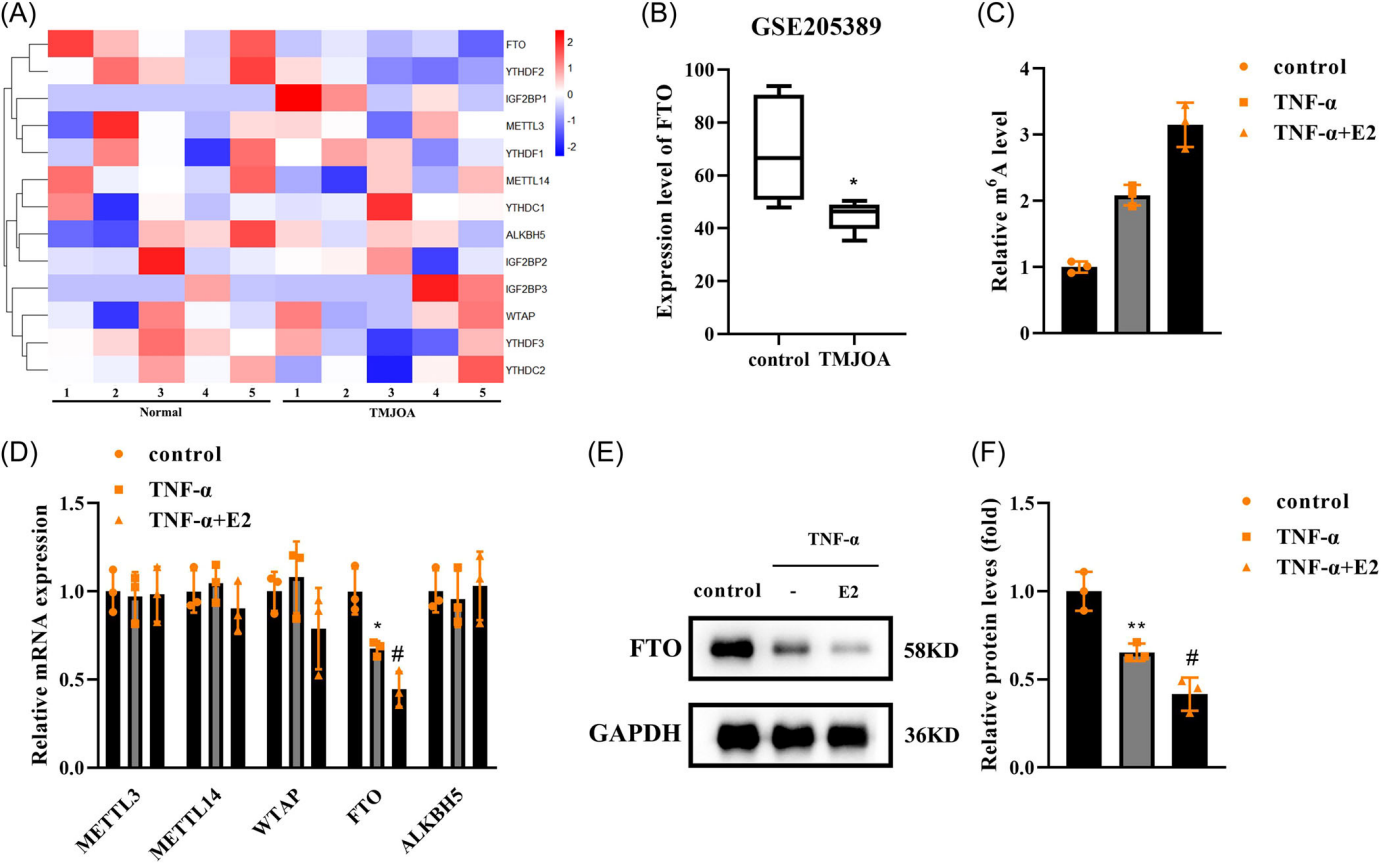
E2 downregulated FTO levels. (A) Differential expression of m6A methylation‐related enzymes in TMJOA was predicted using the GSE205389 microarray and the results showed using a heatmap. (B) The differentially expressed FTO in control and TMJOA. (C) m6A expression was measured in FLSs treated with TNF‐α and E2. (D) qPCR was performed to measure the mRNA expression of METTL3, METTL14, WTAP, FTO, and ALKBH5 in FLSs. (E) Western blot measured the protein levels of FTO in FLSs. (F) FTO protein levels were quantified. *n* = 3. **p < .01. *p < .05. #*p* < .05. FLSs, fibroblast‐like synoviocytes.

To identify the molecular mechanisms of E2, we used bioinformatic analysis to screen the differentially expressed m6A‐related genes (Figure 3A). Among them, FTO expression was significantly decreased in TMJOA (Figure 3B). Then, the m6A level was increased by TNF‐α and was further increased by E2 (Figure 3C). E2 only decreased FTO expression in TNF‐α‐treated FLSs rather than METTL3, METTL14, WTAP, and ALKBH5 (Figure 3D). The western blot experiment also showed that FTO levels were reduced by E2 in TNF‐α‐stimulated FLSs (Figure 3E,F). The results indicated that FTO‐regulated m6A methylation is related to TMJOA.

### 3.4 FTO promotes the demethylation of NLRC5 via IGF2BP1

**Figure 4:**
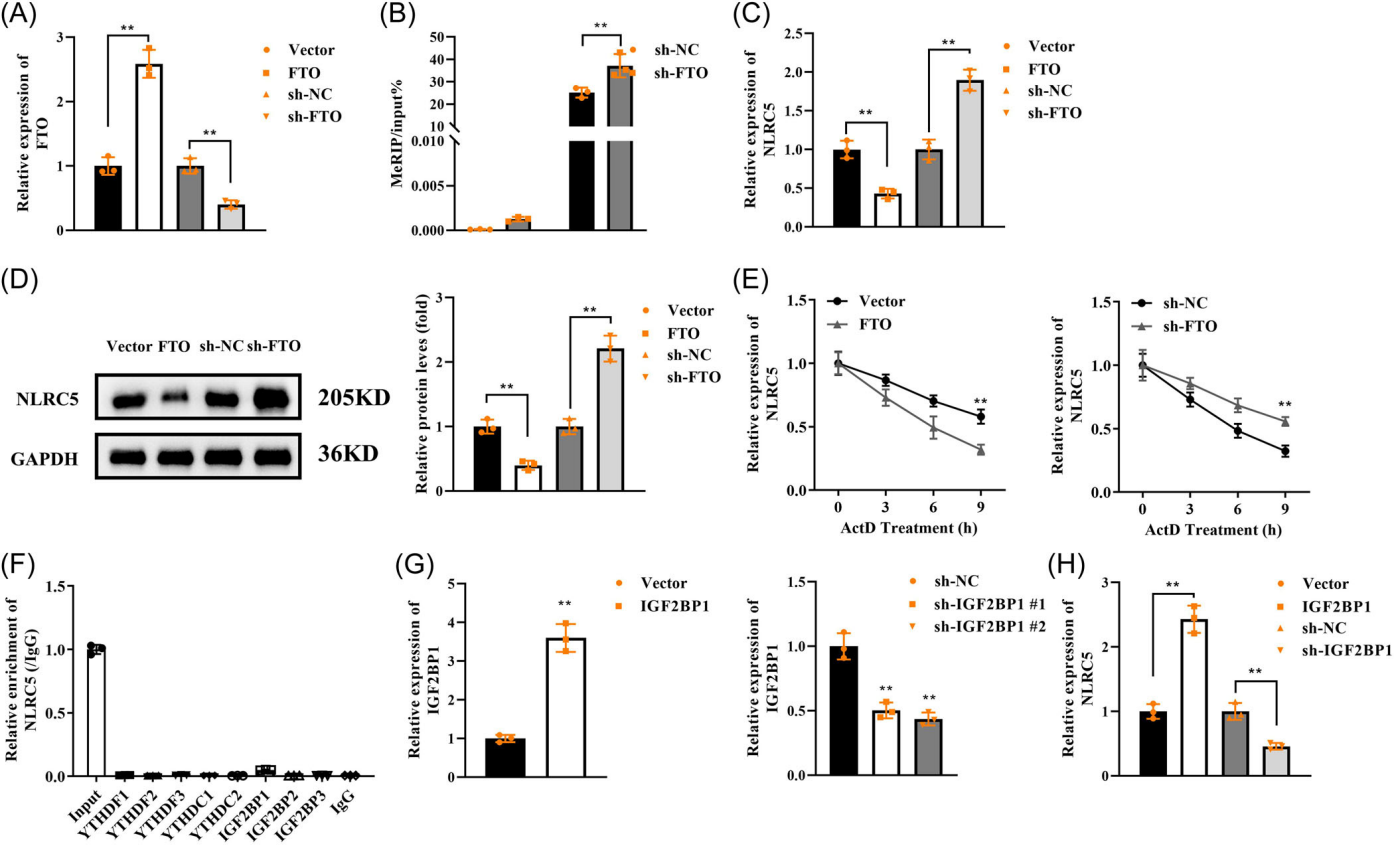
FTO promotes the demethylation of NLRC5 via IGF2BP1. (A) Transfection efficiency was measured using qPCR. (B) MeRIP evaluated the m6A methylation level of NLRC5 regulated by FTO. (C) NLRC5 mRNA expression and (D) protein levels were affected by FTO. (E) mRNA stability of NRC5 was evaluated in transfected FLSs using ActD‐qPCR. (F) The effects of m6A readers on NLRC5 expression were detected by RIP. (G) qPCR was performed to determine IGF2BP1 expression after its overexpression plasmids or sh‐IGF2BP1 transfection. (H) NLRC5 expression was examined by qPCR after IGF2BP1 overexpression or knockdown. *n* = 3. **p < .01.

NLRC5 is a large member of the NLR family that is an inflammatory factor regulating inflammatory responses, immunity, angiogenesis, and apoptosis.^24^ Previous studies have revealed that NLRC5 plays a critical role in rheumatoid arthritis has been found to regulate FLS invasion, migration, and inflammation.^25^, ^26^ Hence, we speculated that NLRC5 may also be involved in TMJOA by regulating FLS behaviors. We first explored whether FTO regulated methylation of NLRC5, the FTO overexpression vector and sh‐FTO were successfully transfected into FLSs (Figure 4A). Then, we observed that knockdown of FTO increased the m6A level of NLRC5 (Figure 4B). Overexpression of FTO decreased NLRC5 levels, whereas FTO knockdown upregulated NLRC5 levels (Figure 4C,D). NLRC5 mRNA stability was suppressed by FTO and was enhanced by sh‐FTO (Figure 4E). Subsequently, m6A readers recognized NLRC5 m6A methylation were identified. The results showed that among them, only IGF2BP1 could bind to NLRC5 (Figure 4F). To measure the effect of IGF2BP1 on NLRC5 expression, IGF2BP1 overexpression plasmids were transfected into FLSs, and its expression was elevated (Figure 4G). Inversely, IGF2BP1 expression was reduced after sh‐IGF2BP1#1 and IGF2BP1#2 transfection, especially IGF2BP1#2 (Figure 4G), which was used in the following experiments. Silencing of IGF2BP1 downregulated NLRC5 expression, whereas overexpressing IGF2BP1 upregulated NLRC5 expression (Figure 4H). Taken together, FTO inhibited m6A methylation of NLRC5 and destructed NLRC5 mRNA stability, which was recognized by m6A reader IGF2BP1.

### 3.5 The effects of E2 on cellular processes are regulated by NLRC5

**Figure 5:**
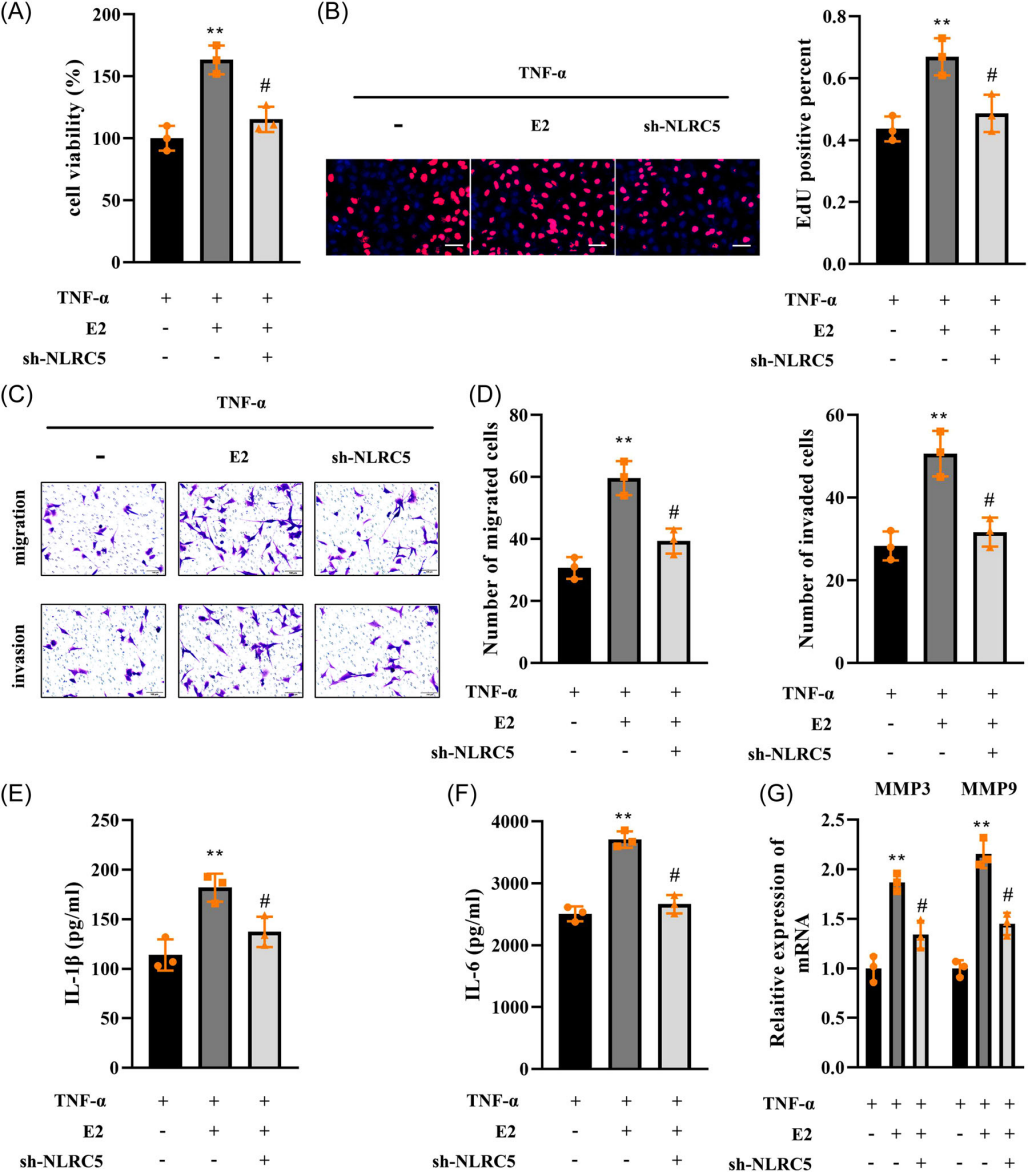
The effects of E2 on cellular processes are regulated by NLRC5. FLSs were transfected with sh‐NLRC5, followed by being treated with TNF‐α and E2, cell proliferation was analyzed by (A) CCK‐8 and (B) EdU assay, scale bar: 100 μm, magnification: ×20; (C and D) Transwell assay evaluated migration and invasion, scale bar: 100 μm, magnification: ×20; (E) IL‐1β and (F) IL‐6 levels were measured by ELISA; and (G) MMP3 and MMP9 expression was detected using qPCR. *n* = 3. **p < .01. #*p* < .05. FLSs, fibroblast‐like synoviocytes.

Then, rescue experiments were performed to confirm the underlying mechanisms. The results showed that E2 promoted the proliferation, migration, and invasion of FLSs treated with TNF‐α, while NLRC5 knockdown abrogated the promotion of cellular processes (Figure 5A–D). Additionally, E2 increased the levels of IL‐1β, IL‐6, MMP3, and MMP9, which were reversed by NLRC5 depletion (Figure 5E–G).

### 3.6 E2 aggravates MIA‐induced TMJOA‐like changes by regulating NLRC5

**Figure 6:**
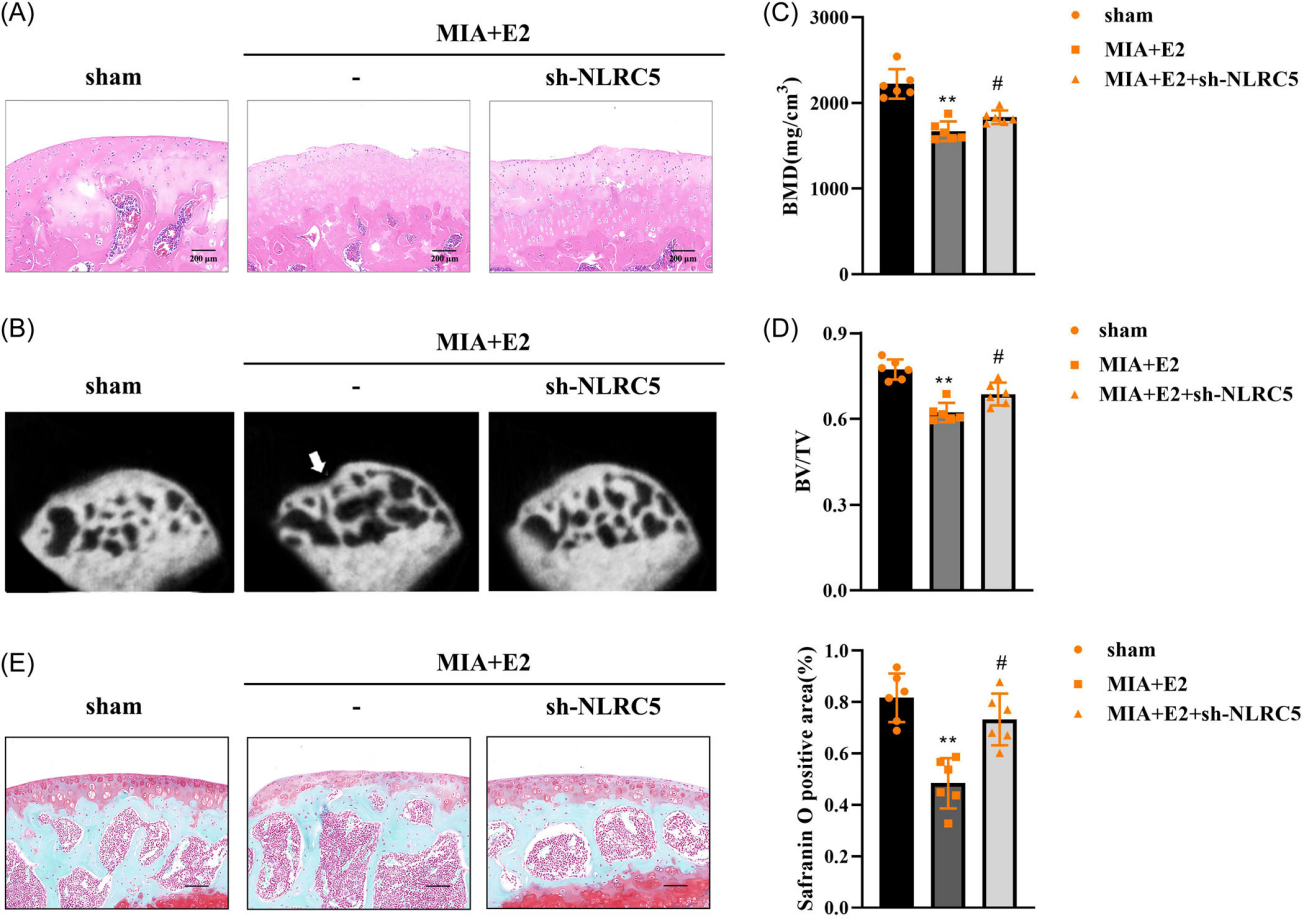
E2 aggravates MIA‐induced TMJOA‐like changes by regulating NLRC5. (A) The histopathology of TMJ condyle was assessed using H&E staining assay. Scale bar: 200 μm, magnification: ×50. (B) The bone microarchitecture of TMJ was analyzed using micro‐CT technology. (C) BMD and (D) BV/TV were quantified in TMJ. (E) Represent images of safranin O staining of condylar tissues. Scale bar: 200 μm. The percentage of safranin O positive area to the whole cartilage layer was quantified. *n* = 6. **p < .01 compared with the sham group. #*p* < .05 compared with the MIA+E2 group.

Finally, we identify the role of E2 and NLRC5 in vivo. We used MIA to establish the TMJOA animal model. The results of H&E staining showed that E2 deteriorated joint pathology and promoted the proliferation of synovial fibroblasts in MIA rats, which were reversed by NLRC5 knockdown (Figure 6A). Additionally, E2 caused disarrangement of trabecular bone in TMJ and subchondral bone surface defection in MIA rats, while NLRC5 knockdown attenuated these adverse effects induced by E2 (Figure 6B). As compared to the sham group, BMD and BV/TV were decreased by MIA and E2. However, silencing of NLRC5 reversed the decrease of BMD and BV/TV in E2‐treated MIA rats (Figure 6C,D). Besides, safranin O staining results showed that the cartilage area was decreased in the MIA+E2 group, compared with the sham group, whereas knockdown of NLRC5 in counteracted the decreased cartilage area in E2‐treated MIA rats (Figure 6E). The results demonstrated that E2 promoted MIA‐induced TMJOA in rats by upregulating NLRC5.

## 4 DISCUSSION

In this study, a TNF‐α‐induced cell model was established to evaluate the effects of E2 on cellular processes. Besides, the mechanisms of E2 action were investigated. Moreover, an MIA‐induced animal model was generated to assess the regulation of E2 on TMJ pathology and bone microstructure.

The sex difference in the incidence of TMJOA suggests a potential role for estrogen in TMJOA, which mainly occurs in women of pubertal and reproductive age.^27^ Estrogen‐sensitized synoviocytes may be an important cause of the gender difference.^11^ Wang and colleagues have demonstrated that E2 administration enhances condylar cartilage degeneration and subchondral bone loss by regulating related estrogen receptors.^19^ Zhao and Gan have indicated that E2 induces apoptosis of chondrocytes, and its combination with hyperlipidemia can promote TMJOA‐like lesions.^18^ In contrast, Wu and colleagues have revealed that estrogen deficiency combined with excessive mechanical stress promotes TMJOA, indicating that estrogen can reduce TMJOA to some extent.^28^ Zhang and colleagues also revealed that a deficiency of E2 results in TMJOA.^29^ Here, we observed that E2 damaged TMJ tissue pathology, promoted subchondral bone surface defection, and decreased BMD and BV/TV, supporting the view that E2 promotes the development of TMJOA. Several studies have shown that synoviocyte proliferation, invasion, and migration are associated with the development of arthritis.^30^, ^31^ Additionally, ECM degradation is related to cartilage destruction induced by TMJOA, and MMP3 and MMP9 are the key drivers of ECM degradation.^32^ Therefore, we considered that E2 aggravated TMJOA by facilitating these phenotypes of FLSs.

FTO expression was downregulated in TMJOA and TNF‐α‐induced FLSs, and E2 treatment further decreased FTO levels, suggesting that E2 regulates cell phenotypes by mediating FTO. FTO is an obesity‐related gene that is associated with metabolic disorders. Importantly, FTO is a demethylase that regulates m6A methylation modification.^33^ m6A methylation is the most prevalent RNA modification that regulates the development of multiple diseases, including the regulation of the immune microenvironment in OA.^34^, ^35^ m6A methylation is mediated by methyltransferase complex, demethylases, and methylated reading proteins, which act as “writers,” “erasers,” and “readers,” respectively.^36^ Based on the demethylation effect of FTO, it is natural for us to think that FTO regulates the m6A methylation modification of genes. NLRC5 is a transactivator of the MHC class I molecular family, which is associated with immune responses.^37^ In inflammation‐related diseases, NLRC5 is highly expressed in immune cells and tissues.^38^ Mu and colleagues, have reported that NLRC5 can inhibit the production of a variety of inflammatory factors and reduce OA.^39^ In contrast, dexmedetomidine suppressed rheumatoid arthritis by downregulating NLRC5 expression.^25^ These studies show that it is controversial whether NLRC5 plays a pro‐inflammatory or anti‐inflammatory role. Whether NLRC5 is involved in TMJOA by m6A methylation has not been reported. In the current study, we observed that knockdown of FTO promoted m6A methylation levels of NLRC5, enhanced its mRNA stability, and negatively regulated NLRC5 levels. The data suggest that NLRC5 has inflammation‐promoting effects in TMJOA. E2 promotes TMJOA development through FTO‐regulated m6A demethylation of NLRC5.

Additionally, the m6A reader was identified. We found that only IGF2BP1 could bind to NLRC5 and positively regulate NLRC5 expression. IGF2BP1 is a cytoplasmic m6A reader that can enhance mRNA stability by the combination of m6A motif and target transcripts.^40^, ^41^ Hence, we considered that IGF2BP1 stabilized NLRC5 in an m6A‐dependent manner. Taken together, FTO inhibited m6A methylation of NLRC5 and reduced the stability dependent on the IGF2BP1 reader.

However, limitations are existed in this study. For example, the methylation sites in NLRC5 remain unknown. In addition, we have not yet evaluated the effect of E2 and NLRC5 on TMJ or even systemic inflammation in vivo. The changes of synoviocytes in vivo affected by E2 and NLRC5 are also not been investigated. We will further resolve these limitations in our future work.

In conclusion, we found that E2 regulated NLRC5 to accelerate the progression of TMJOA by facilitating the proliferation, migration, invasion, inflammation, and ECM degradation of FLSs. Mechanically, E2 downregulated FTO, which promotes the m6A demethylation of NLRC5 in the IGF2BP1‐dependent manner. These findings provide a theoretical basis for E2 to accelerate the progress of TMJOA.

## AUTHOR CONTRIBUTIONS

All authors participated in the design, interpretation of the studies and analysis of the data and review of the manuscript. Xintong Xue drafted the work and revised it critically for important intellectual content; Changyi Li, Shuang Chen, Yan Zheng, and Fan Zhang were responsible for the acquisition, analysis, or interpretation of data for the work; Yan Xu made substantial contributions to the conception or design of the work. All authors read and approved the final manuscript.

## CONFLICT OF INTEREST STATEMENT

The authors declare no conflict of interest.

## ETHICS STATEMENT

The animal study was approved by the Ethics Committee of Shanghai Stomatological Hospital, Fudan University and was performed according to the ARRIVE guidelines.

## Data Availability

The data sets used and/or analyzed during the current study are available from the corresponding author on reasonable request.
